# Injury to the Endothelial Surface Layer Induces Glomerular Hyperfiltration Rats with Early-Stage Diabetes

**DOI:** 10.1155/2014/953740

**Published:** 2014-04-09

**Authors:** Chunyang Zhang, Yao Meng, Qi Liu, Miao Xuan, Lanyu Zhang, Bo Deng, Keqin Zhang, Zhimin Liu, Tao Lei

**Affiliations:** ^1^Division of Endocrinology, Shanghai Tongji Hospital Affiliated Tongji University, No. 389, Xincun Road, Shanghai 200065, China; ^2^Key Laboratory of Nutrition and Metabolism, Institute for Nutritional Sciences, Shanghai Institutes for Biological Sciences, Chinese Academy of Sciences, Shanghai 200031, China; ^3^Department of Endocrinology, Changzheng Hospital, Second Military Medical University, Shanghai 200041, China

## Abstract

Glomerular endothelial surface layer (ESL) may play a role in the mechanisms of albuminuria in diabetic nephropathy, which lack evidence *in vivo*. The effects of high glucose on the passage of albumin across the glomerular ESL were analysed in streptozotocin-induced diabetic Sprague-Dawley rats for 4 weeks. Albuminuria and glomerular mesangial matrix were significantly increased in diabetic rats. The passage of albumin across the ESL, as measured by albumin-colloid gold particle density in the glomerular basement membrane (GBM), was increased significantly in diabetic rats. The thickness of the glomerular ESL, examined indirectly by infusing Intralipid into vessels using an electron microscope, was significantly decreased and the GBM exhibited little change in diabetic rats. In summary, the glomerular ESL may play a role in the pathogenesis of albuminuria in rats with early-stage diabetes.

## 1. Introduction

Albuminuria is a sign of kidney disease, including diabetic nephropathy. The glomerular capillary wall acts as a glomerular filtration barrier (GFB) and includes 3 components: fenestrated glomerular endothelium, glomerular basement membrane (GBM), and podocytes with processes bridged by slit diaphragms [1]. The effects of the GBM and podocytes on the permselective properties of the GFB have attracted much interest in recent years; however, the contribution of the endothelium has received less attention [2]. Because the fenestrae of glomerular endothelial cells are approximately 60 nm diameter without the diaphragm and albumin has a diameter of only 3.6 nm, the endothelium is thought to contribute little to the protein barrier function of the glomerular capillary wall.

Recently, several lines of evidence have suggested that there is a 200- to 400-nm-thick membrane-like layer covering the luminal surface of the glomerular endothelium; this is called the endothelial cell surface layer (ESL) [3, 4]. The ESL is a hydrated, gel-like structure that includes 2 components: the glycocalyx, which is connected to the endothelium with several “backbone” molecules, mainly proteoglycans and glycoproteins, and the endothelial cell coat, which is attached to the glycocalyx and is composed of secreted proteoglycans, glycosaminoglycans, glycoproteins, and plasma proteins [1, 5, 6].

Many studies have shown that the ESL also plays an important role in the permeability barrier (including the GFB) [7, 8]. Patients with preeclampsia or hemolytic uremic syndrome have albuminuria, and their glomerular endothelia are injured [9, 10]. Additionally, Jeansson and Haraldsson showed that infusion enzymes, which digest glycosaminoglycans, decrease the thickness of the ESL and promote increased flux of albumin across the glomerular capillary barrier [11, 12]. Singh et al. demonstrated that treatment with heparanase on the human glomerular endothelial cell glycocalyx* in vitro* removed the majority of the glycocalyx and increased the passage of albumin across the cell monolayer [13]. These studies suggested that injuries to the glomerular ESL increased the flux of albumin across the GFB and caused proteinuria, indicating the importance of the ESL in glomerular permselectivity.

Microalbuminuria has emerged as an important risk factor for the development of cardiovascular disease and a marker of diabetic nephropathy. The pathogenesis of microalbuminuria in patients with diabetes is not fully understood. However, studies have shown that endothelial dysfunction is present well before the onset of microalbuminuria in type 1 diabetes [14]. Additionally, reduced systemic glycocalyx volume is inversely correlated with the albumin-to-creatinine ratio in type 1 diabetes [15, 16]. High glucose reduces the biosynthesis of glycosaminoglycan in the glomerular endothelial cell glycocalyx and increases the passage of albumin across endothelial cell monolayers [17]. Studies have shown that hyperglycemia leads to reduced charge-selectivity and proteinuria, altering the composition of the glomerular barrier [18]. However, to fully elucidate the mechanisms involved in these processes, we must first determine whether ESL plays a role in the protein barrier function of the GFB* in vivo*.

In this study, we investigated the passage of albumin across the glomerular barrier, especially the glomerular ESL and GBM, using electron microscopy. We also examined changes in the 2 layers of the GFB in early diabetic rats. Our data have important implications in the role of the glomerular barrier in diabetes.

## 2. Research Design and Methods

### 2.1. Induction of Diabetes and Experimental Protocols

The experimental protocol was approved by the Animal Ethics Review Committee of Shanghai Institutes for Biological Sciences in the Chinese Academy of Sciences. Male Sprague-Dawley rats were fed with a standard laboratory diet and were provided with water ad libitum. Experimental rats, weighing approximately 200 g, received an intraperitoneal injection of streptozotocin (STZ, Sigma, St. Louis, MO, USA, 60 mg/kg body wt) dissolved in 0.1 mol/L sodium citrate, pH 4.6. Control rats (*n* = 6) received injection with buffer alone. Six days after the injection of STZ, rats with blood glucose levels above 16.7 mmol/L were included in the diabetes group (*n* = 6).

Blood glucose and body weight were monitored every week. No rats died during the study, and no signs of apparent exhaustion were observed during the experimental period. Five weeks after the injection of STZ, individual 24-hour urine sample collections were performed using metabolic cages. The rats were anaesthetized and the kidneys were harvested and weighted. Then the animals were sacrificed.

### 2.2. Urine Examination

Urine was collected and stored at 4°C until analysis. Albumin concentrations were determined using an enzyme-linked immunosorbent assay (ELISA) kit (RayBiotech, Inc., Norcross, GA, USA). The absorbance of each well of the ELISA plate was measured using a Microplate reader (SpectraMax Plus384, Molecular Devices, USA) at 450 nm. The concentration of urinary albumin was calculated using a standard curve.

### 2.3. Histological Analysis

At 4 weeks after the initiation of treatment, kidneys were removed, fixed in 10% buffered formalin, and embedded in paraffin. Sections were stained with hematoxylin and eosin (HE) for observation by light microscopy. Ten glomeruli in the cortex were examined per animal under high magnification (400×). The glomerular tuft area and mesangial matrix area were measured by manually tracing the glomerular tuft and mesangial matrix using Image-Pro Plus software. The mesangial matrix index represented the ratio of mesangial matrix area divided by the tuft area. The results are expressed as means ± standard deviations (SDs).

### 2.4. Immunocytochemistry

The kidney cortex was cut into 1 mm^3^ pieces and fixed in 0.5% glutaraldehyde/4% paraformaldehyde in 0.1 M phosphate buffer (pH 7.2) at 4°C for 2 h. Tissues were then rinsed in 0.1 M phosphate buffer. Specimens were dehydrated in a graded series of ethanol (70%, 80%, and 95%). Infiltration with LR White resin was carried out for 24 h, and tissues were embedded in gelatin capsules. Polymerization of the resin was carried out by the addition of LR White accelerator at −20°C for 5 days. Ultrathin sections (50–60 nm) were obtained with a LKB-I ultracut microtome and were collected on 200-mesh nickel support grids.

Thin sections were sequentially quenched for 1 h at room temperature (RT) in phosphate-buffered saline (PBS) containing 1% bovine serum albumin (BSA), incubated for 2 h at RT with anti-albumin antibodies (Abcam Ltd, USA) diluted 1 : 150 in A-PBS, and washed 3 times for 15 min each with PBS. Sections were then incubated with gold-tagged (6 nm) anti-rabbit IgG (Abcam Ltd.) diluted 1 : 20 in PBS and fixed for 10 min with 2.0% GA. After staining with uranyl acetate, the sections were examined under a HITACHI H-7650 electron microscope operating at 80 kV.

The specificity of each immunolabeling assay was assessed by various control experiments: incubation with gold-tagged (6 nm) anti-rabbit IgG alone, omitting the antibody, and incubation with the antibody solution containing excess antigen, followed by analysis with the gold-tagged antibody.

Micrographs were obtained from 10–20 sections of the GBM (a total length of 40 *μ*m) per rat. Images (final magnification of 20,000×) were scanned and analyzed using Image-Pro Plus software. For each section of the GBM, the number of albumin particles was manually counted. The results were expressed as particle densities per unit length (*μ*m).

### 2.5. Experiments with Intralipid

Intralipid (Sino-Swed Pharmaceutical Corp., Ltd., Beijing) was centrifuged for 10 min at 3000 rpm to obtain enriched floating lipid-particle fractions. Then, 1.5 mL of each fraction was injected into the inferior caval vein by puncturing with a thin needle. The lipid emulsion was allowed to mix with the circulating blood for 10 min, and the kidneys were then removed and fixed in 2.5% glutaraldehyde in 0.1 M phosphate buffer (pH 7.2) for 2 h at 4°C. All specimens were postfixed in 1% OsO_4_ for 2 h at 4°C and dehydrated through increasing concentrations of ethanol (50%, 70%, and 90%) for 20 min. Samples were then embedded in Epon and were heat cured. Ultrathin sections (50–60 nm) were obtained with a LKB-I ultracut microtome and were collected on 200-mesh nickel support grids. Sections were contrasted with lead citrate and uranyl acetate and examined under a HITACHI H-7650 electron microscope.

Micrographs were obtained from 5 randomly selected glomerular capillaries per rat. Images (final magnification of 8,000×) were scanned and analyzed using Image-Pro Plus software. The capillary luminal area was divided into 2 regions: (1) the peripheral region defined as extending 200 nm from the luminal surface of the endothelium and (2) the central region. For each glomerular capillary, the areas of these 2 regions were calculated, and the number of lipid particles was manually counted. The results were expressed as particle density per unit area (*μ*m^2^).

### 2.6. Morphometric Analysis of the GBM

Micrographs were obtained from 5 randomly selected glomerular capillaries per rat. GBM thickness was measured in electron micrographs, where the thickness was defined as the distance between the podocyte process and the corresponding endothelial cell. To ensure proper cross-sectioning, a slit diaphragm between the processes had to be visible and a single layer of endothelial cells had to be present.

### 2.7. Statistical Analysis

Results are presented as means ± standard deviations (SDs). Student's *t* tests were used to compare data between 2 groups. Differences with *P* values of less than 0.05 were considered statistically significant.

## 3. Results

### 3.1. Changes in Blood Glucose and Body Weight

Plasma glucose concentrations were significantly higher in diabetic animals than in nondiabetic animals (23.9 ± 2.8 mmol/L versus 3.67 ± 0.64 mmol/L, resp.). Body weight was significantly lower in the diabetic group than in the nondiabetic group (272 ± 27.4 g versus 466 ± 31.8 g, resp.).

### 3.2. Changes in Urinary Albumin Excretion

**Figure 1:**
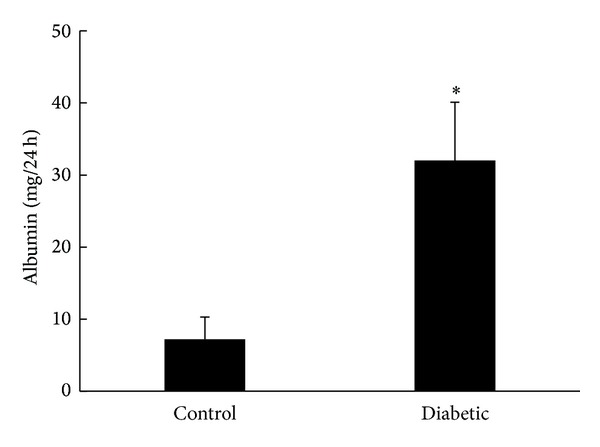
Twenty-four UAE in diabetic rats versus control rats.*P < 0.01.

To evaluate glomerular hyperfiltration induced by hyperglycemia in diabetic rats, we measured 24-hour urinary albumin excretion (UAE, Figure 1). UAE was significantly higher in diabetic rats than in nondiabetic control rats.

### 3.3. Histology and Morphometric Analysis

**Figure 2:**
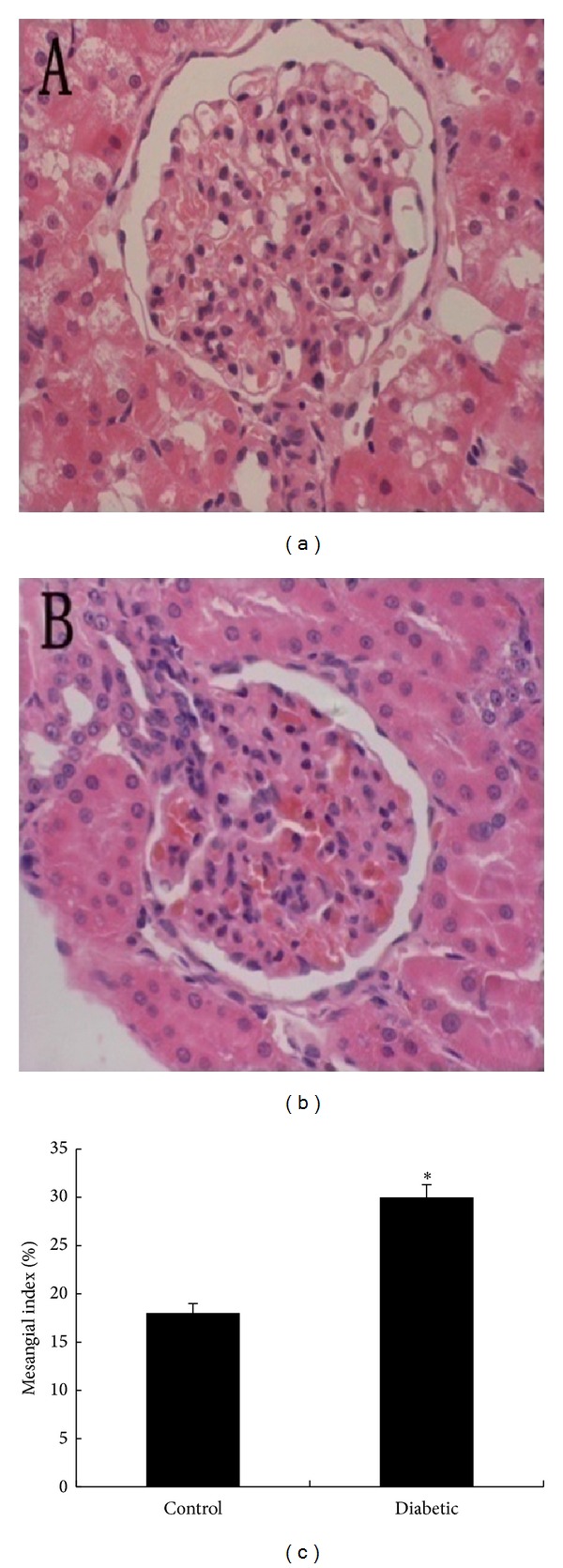
(a)-(b) Representative light microscopy images of glomeruli (original magnification, 400×) in nondiabetic control rats (a) and diabetic rats (b). (c) Mesangial index in control and diabetic rats, expressed quantitatively by calculating the percentage of the total glomerular area. *P < 0.01 versus (c) *n* = 6 for each group. Each column represents the mean ± SD.

Morphometric analysis of glomeruli revealed that hyperglycemia in diabetic rats significantly increased mesangial matrix accumulation (mesangial matrix area/glomerular tuft area) compared with that observed in control rats (Figure 2).

### 3.4. Transendothelial Albumin Permeability

**Figure 3:**
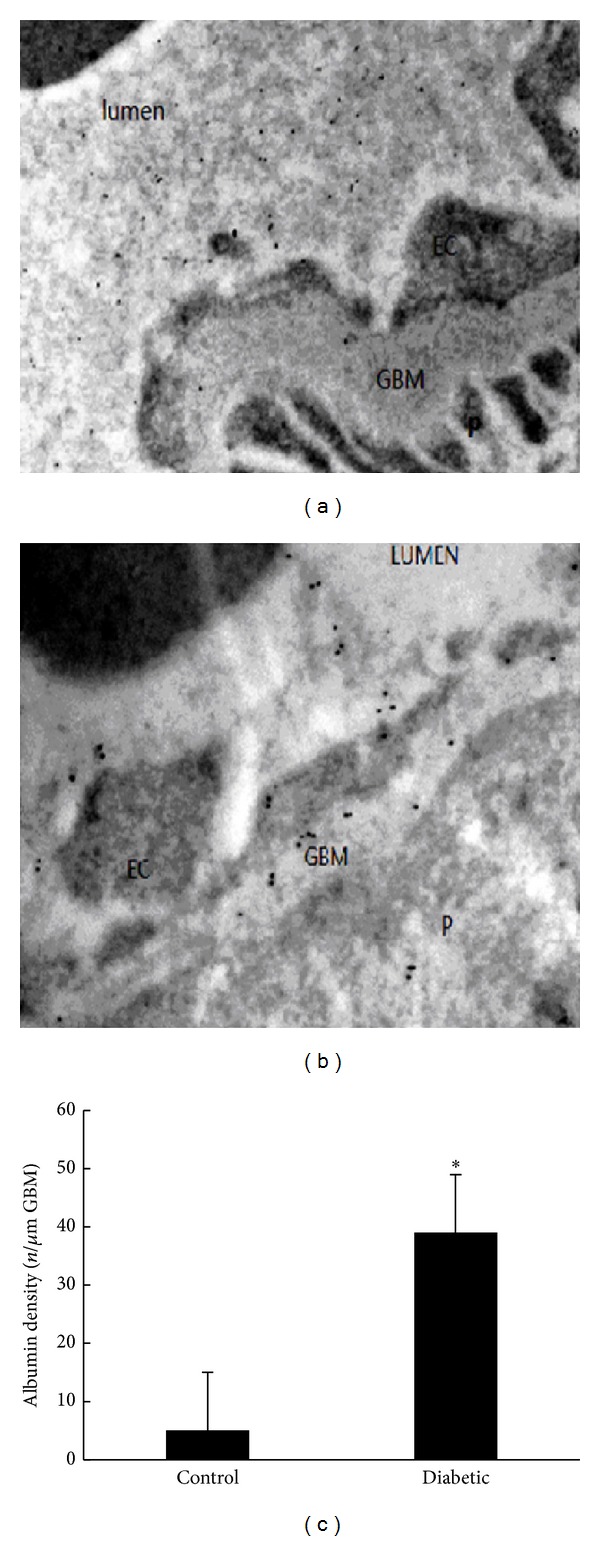
(a)–(c) Increased GFB permeability to albumin in diabetic rats, as revealed by postembedding immunocytochemistry. (a) Control rats; (b) diabetic rats; (c) increased albumin in the GBM of diabetic rats. *P < 0.05 versus (c).

We next used albumin-colloid gold (A-CG) as a tracer to quantify changes in albumin transport. The permeability of A-CG was quantified as described in the Methods Section. As shown in Figure 3, in control rats, the tracer was located mainly over the glomerular capillary lumen, and the GBM and podocytes were almost completely free of labeling. In diabetic rats, many more gold particles were observed in the GBM and podocytes than in control rats.

### 3.5. Experiments with Intralipid

**Figure 4:**
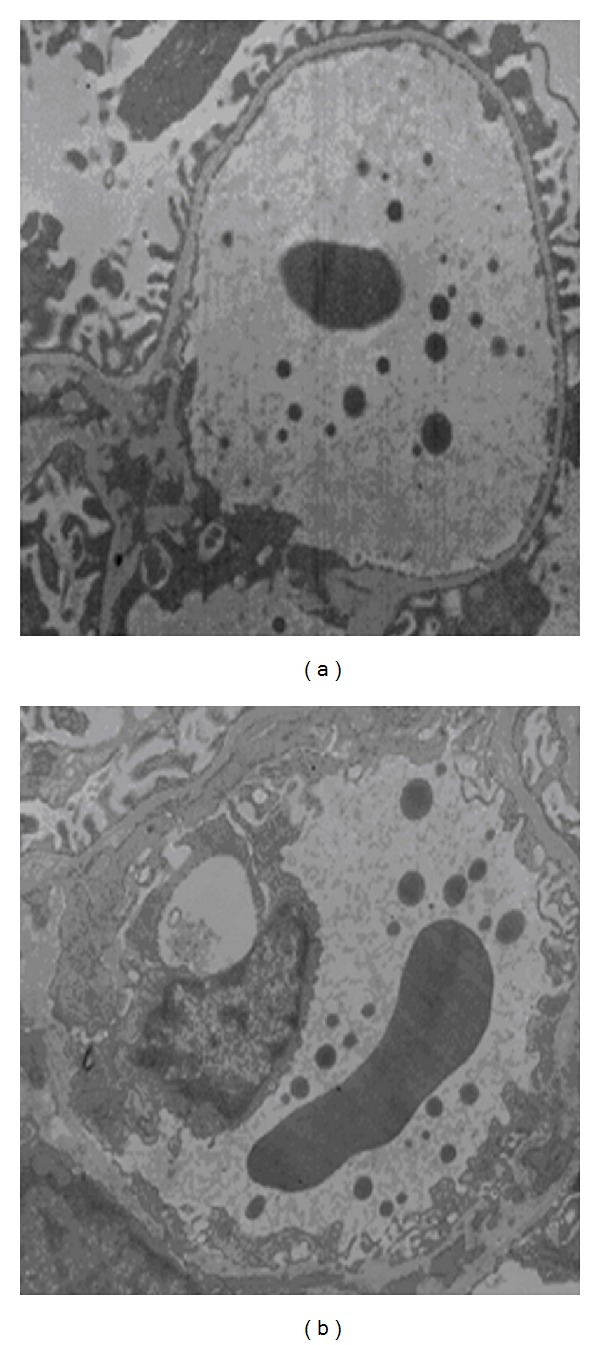
Indirect examination of the thickness of the glomerular endothelial surface layer. Kidneys were fixed in glutaraldehyde after the addition of Intralipid particles to the circulation. (a) Control and (b) diabetic rats.

**Figure 5:**
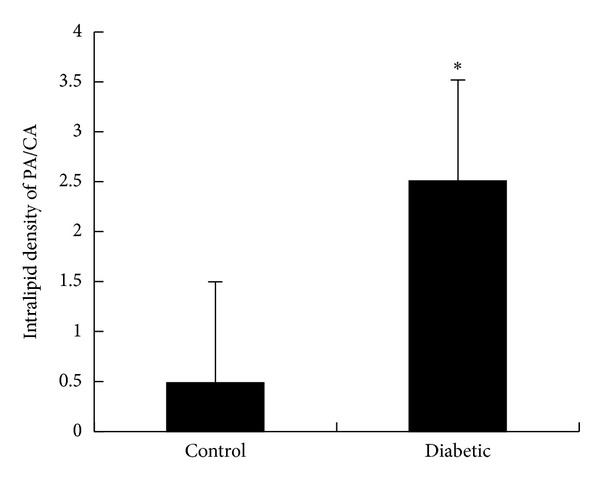
Ratio of Intralipid particle density between the PA and CA of the glomerular capillaries (*P < 0.01). Results from *n* = 6 rats; 5 glomerular capillaries were examined in each rat. PA: periendothelial area and CA: central area.

Lipid particles were easily recognized on the micrographs as spherical bodies of medium electron density, seemingly randomly distributed in the glomerular capillary lumen (Figure 4). The lipid-particle density was significantly lower in the 200-nm periendothelial region than in the central region of the glomerular capillaries in control rats (see Figure 5). Compared with control rats, there were significantly more lipid particles in the 200-nm periendothelial region of the glomerular capillary lumen in diabetic rats (see Figure 5, *P* < 0.01).

### 3.6. Morphometric Analysis of the GBM

**Figure 6:**
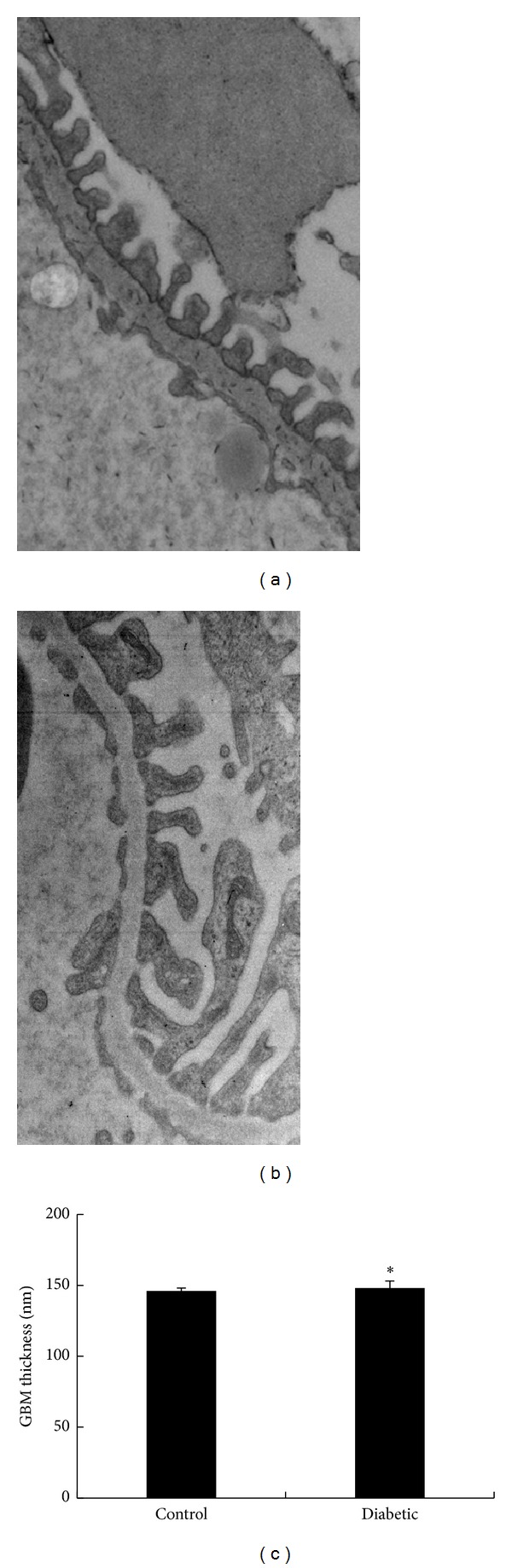
Morphological changes in the GBM of the glomerulus. (a) Control rats, (b) diabetic rats, and (c) thickness of the GBM (*P > 0.05).

The thickness of the GBM did not differ significantly between control and diabetic rats (146 ± 2 nm versus 148 ± 5 nm, respectively; see Figures 6(a)–6(c)).

## 4. Discussion

In the present study, a diabetic rat model was induced by STZ and changes in blood glucose, body weights, and glomerular filtration were analyzed. Our results showed that blood glucose in diabetic rats was higher than that in control rats. Additionally, the body weights of diabetic rats were lower than those of control rats. Although diabetic rats exhibited significant weight loss, they failed to show any signs of apparent exhaustion during the experimental period.

Microalbuminuria is a characteristic change observed in early diabetic nephropathy. Therefore, we chose to analyze diabetic rats at 4 weeks after STZ induction in this study. To evaluate glomerular hyperfiltration induced by hyperglycemia in diabetic rats, we measured 24-hour urinary albumin excretion (UAE). Our results showed that the UAE of diabetic rats was higher than that of control rats, indicating that hyperglycemia increased the glomerular filtration of diabetic rats at 4 weeks after injection of STZ.

The pathological features of diabetic nephropathy, as observed under a light microscope, include expansion of the mesangial region and extracellular matrix. Our study showed similar changes in glomerular morphology in diabetic rats, indicating that hyperglycemia damaged the glomeruli at 4 weeks after STZ. However, these data do not explain the cause of albuminuria.

The exact mechanism underlying proteinuria is not well understood. A popular hypothesis is that layers of the glomerular capillary wall must be viewed as an integrated ultrafilter, in which defects in any of the 3 glomerular layers may result in proteinuria [1]. Moreover, examination of glomeruli under an electron microscope showed that there were few histological changes in the endothelium and GBM between diabetic and control rats. Quantitative analysis of the GBM revealed that differences in GBM thickness between diabetic and control rats were minimal. Therefore, we believe that hyperglycemia may have caused minimal changes in the endothelium and GBM in diabetic rats. However, it seems unlikely that albuminuria may be caused by such minimal changes in the endothelium and GBM in diabetic rats.

It is unclear whether glomerular ESL injury caused albuminuria in diabetic rats and little direct* in vivo* evidence is available to confirm or refute this hypothesis. The ESL is a complicated structure to study; it is easily dehydrated or disrupted and hard to visualize. As previously shown, functional data indicate that the glomerular capillary wall has a fixed negative charge density of 35–40 mEq/L [19]. The small lipid particles of Intralipid carry a negative charge at physiological pH. Thus, the lipid-particle exclusion zone could reflect electrostatic interactions with an assumed ESL formed by a negatively charged polysaccharide-rich gel. Concerning measurement of the thickness of glomerular ESL, detection with electron microscopy after injection of Intralipid into kidney blood vessels had little influence on ESL and can better show the real thickness of ESL among all the detection approaches [4]. Therefore, we measured the thickness of the glomerular ESL indirectly using electron microscopy after injection of Intralipid into the veins of our experimental rats. Our results showed that the frequency of Intralipid droplets found 0–200 nm from the endothelial cell membrane increased significantly in diabetic rats compared with control rats. These data indicated that the ESL was injured in diabetic rats and that more Intralipid droplets entered regions covered by glycocalyx. The mechanisms of glomerular ESL injury by hyperglycemia are not clear. Excessive generation of reactive oxygen species (ROS) is central to the pathogenesis of diabetic nephropathy [20]. A recent study showed that ROS directly alters the critical components of the glomerular endothelial cell glycocalyx and that these changes have implications in the barrier function of this region, affecting the passage of albumin* in vitro* [21].

Finally, we also measured endogenous albumin passage across the GFB by immune electron microscopy. We found a significant increase in albumin entry into the GBM through the ESL and endothelial cells in diabetic rats, indicating that the permeability of the ESL increased. These findings were consistent with the aforementioned glomerular ESL injury. Thus, we believe that the increased permeability of the GFB and the augmentation of albuminuria may be induced by ESL injury in early diabetic rats.

In summary, we present both morphological and functional evidence that injury to the ESL changed the permeability of the glomerular barrier and induced albuminuria in early diabetes. However, significant injury to the GBM has yet to be observed.
